# Nonlinearity of Mechanochemical Motions in Motor Proteins

**DOI:** 10.1371/journal.pcbi.1000814

**Published:** 2010-06-17

**Authors:** Yuichi Togashi, Toshio Yanagida, Alexander S. Mikhailov

**Affiliations:** 1 Nanobiology Laboratories, Graduate School of Frontier Biosciences, Osaka University, Suita, Osaka, Japan; 2 Department of Physical Chemistry, Fritz Haber Institute of the Max Planck Society, Berlin, Germany; D.E. Shaw Research, United States of America

## Abstract

The assumption of linear response of protein molecules to thermal noise or structural perturbations, such as ligand binding or detachment, is broadly used in the studies of protein dynamics. Conformational motions in proteins are traditionally analyzed in terms of normal modes and experimental data on thermal fluctuations in such macromolecules is also usually interpreted in terms of the excitation of normal modes. We have chosen two important protein motors — myosin V and kinesin KIF1A — and performed numerical investigations of their conformational relaxation properties within the coarse-grained elastic network approximation. We have found that the linearity assumption is deficient for ligand-induced conformational motions and can even be violated for characteristic thermal fluctuations. The deficiency is particularly pronounced in KIF1A where the normal mode description fails completely in describing functional mechanochemical motions. These results indicate that important assumptions of the theory of protein dynamics may need to be reconsidered. Neither a single normal mode nor a superposition of such modes yields an approximation of strongly nonlinear dynamics.

## Author Summary

Biological cells use a variety of molecular machines representing enzymes, ion channels or pumps, and motors. Motor proteins are nanometer-size devices generating forces and actively moving or rotating under the supply of chemical energy through ATP hydrolysis. They are crucial for many cell functions and promising for nanotechnology of the future. Although such motors represent single molecules, their operation cycles cannot be followed in detail in simulations even on the best modern supercomputers and some approximations need to be employed. It is often assumed that conformational dynamics of motor proteins is well described within a linear response approximation and corresponds to excitation of normal modes. We have checked this assumption for two motor proteins, myosin V and kinesin KIF1A. Our results show that, while both these biomolecules respond by well-defined motions to energetic excitations, these motions are essentially nonlinear. The effect is particularly pronounced in KIF1A where relaxation proceeds through a sequence of qualitatively different conformational changes, which may facilitate complex functional motions without additional control mechanisms.

## Introduction

Protein machines, which may represent enzymes, ion pumps or molecular motors, play a fundamental role in biological cells and understanding of their activity is a major challenge. Operation of these machines is based on slow conformational motions powered by external energy supply, often with ligands (such as ATP). In molecular motors, binding of ATP and its subsequent hydrolysis induce functional mechanochemical motions, essential for their operation. These motions, which follow after an energetic activation, are conformational relaxation processes.

Large-scale conformational changes may take place in proteins as a result of ligand binding [1]. Despite the large magnitude of such changes, they are nonetheless often considered in the framework of the linear response theory [2] and the normal mode approximation [3]–[7]. The normal mode analysis is furthermore broadly employed in the elastic-network studies of proteins [7]–[18]. However, there is no general justification to assume that relaxation processes in proteins are linear and this assumption has to be verified for particular macromolecules.

It is known that relaxation processes in complex dynamical systems may be strongly nonlinear and deviate much from simple exponential relaxation. As an example borrowed from a distant field, we can mention the Belousov-Zhabotinsky reaction which exhibits a great variety of spatiotemporal patterns (pacemakers, rotating spiral waves) that are however only complicated transients accompanying relaxation to the equilibrium state [19], [20]. Conformational relaxation in single protein molecules may also be a complicated process, comprising qualitatively different kinds of mechanochemical motions.

While partial unfolding and refolding, associated with ligand binding, are known for some protein machines, such as the enzyme adenylate kinase [21], usually functional conformational motions in molecular machines and, specifically, in motor proteins are *elastic*. This means that the pattern of contacts between the residues in a protein is not changed upon ligand binding and preserved during the relaxation process, as generally assumed in the elastic network modeling (ENM).

Here, we provide detailed analysis of conformational relaxation processes, associated with ligand binding and hydrolysis, in two motor proteins — myosin V [22], [23] and kinesin KIF1A [24]. Our investigations, performed in the framework of the ENM approximation, reveal that nonlinearity is characteristic for both macromolecules and the normal mode description is not really applicable for any of them. For KIF1A, a monomeric motor protein from the kinesin superfamily, nonlinear effects are found to dominate completely functional mechanochemical motions which turn out to be qualitatively different from the normal mode predictions. Despite the nonlinearity, well-defined conformational relaxation paths, robust against perturbations, have been found in both motor proteins.

## Results

Within the coarse-grained ENM approach, a protein is modeled as a network of point-like particles, corresponding to residues, which are connected by a set of elastic links [9], [10]. A link between two particles is present if the distance between them in the equilibrium conformation of the considered macromolecule is shorter than a cutoff length. The elastic energy of the network is 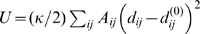, where 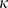 is the stiffness constant of the network links, 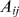 is the matrix of connections inside the network, 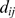 is the distance between particles 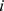 and 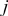, and 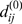 is the respective distance in the equilibrium reference state. The characteristic time scales of functional mechanochemical motions in motor proteins are in the millisecond range and slow conformational relaxation motions on such timescale should be overdamped [25]. Neglecting hydrodynamic interactions, relaxation dynamics is then described by equations 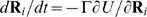 for the coordinates 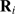 of the particles, where 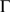 is their mobility. Relaxation dynamics for elastic networks of proteins has been previously considered [26].

Despite a wide-spread misunderstanding, elastic dynamics is generally nonlinear. For example, macroscopic objects, such as ribbons or membranes, can still exhibit pronounced nonlinear effects of spontaneous twisting or buckling, while fully retaining their elastic behavior and not undergoing plastic deformations [27]. The energy 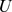 of an elastic network is quadratic in terms of the distances 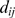 and the forces acting on the particles are linear in terms of such distances. However, the distance 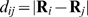 is itself a *nonlinear function* of the coordinates 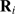 and 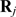 and this makes the forces also nonlinear functions of dynamical variables. The presence of nonlinear effects in conformational relaxation of proteins in the ENM approximation has been previously demonstrated [28], [29].

Explicitly, relaxation dynamics of considered proteins is described by equations (3) in the Methods section, where further details are also given. To study conformational relaxation, these equations were numerically integrated starting from various initial conditions.

### Myosin V

**Figure 1:**
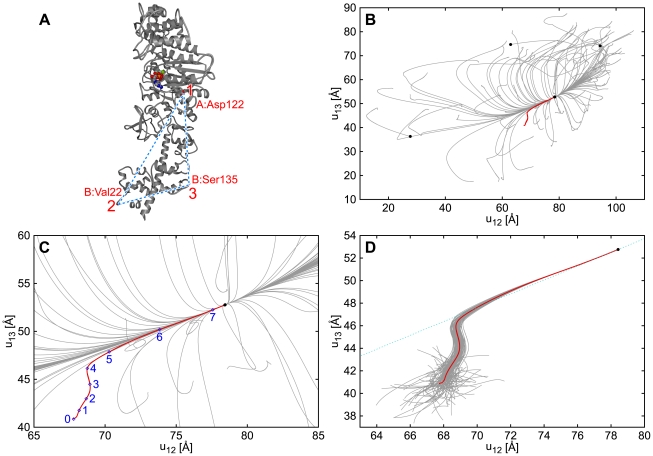
Relaxation paths of myosin V. The elastic network model is constructed for the ATP-bound structure as the reference state. The red line shows the trajectory in the plane of distances 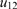 and 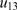 between labels 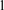, 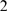 and 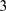 indicated in panel (A) starting from the nucleotide free state, so that this path corresponds to the conformational transition upon ATP binding. In panels (B) and (C) (magnified), gray lines display trajectories starting from 100 different random initial conditions (see Methods). In panel (D), gray lines represent relaxation trajectories with 100 different random deformations applied to the same initial structure as that for the red line. The dotted line in panel (D) shows the direction of distance changes corresponding to the slowest normal relaxation mode. Black dots indicate (meta)stable states reached. Times 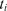 at points 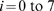 indicated in panel (C) are 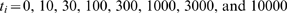, respectively.

The reference conformation, used to construct the elastic network, was that of the ATP(analog) bound state (Protein Data Bank (PDB) ID code: 1W7J, with MgADP-BeFx as the ATP analog [30]). As the initial condition, the conformation corresponding to the nucleotide-free state was taken (PDB ID: 1OE9 [31]). The elastic network had 855 particles connected by 7261 links. Note that only the residues whose 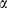-carbon positions are contained in both PDB data sets have been taken to construct the network. Additionally, relaxation processes starting from randomly generated initial conditions (see Methods) have been considered. For visualization purposes, motions of three particles (Asp122 in chain A, and Val22 and Ser135 in chain B) have been traced (Figure 1A). Thus, each relaxation process was characterized by a certain trajectory in the space of distances between the three chosen labels.

Figures 1B,1C display 100 conformational relaxation trajectories, each starting from a different random initial condition. Although the initial conditions were generated by applying relatively strong deformations (without unfolding) to the reference state, almost all of them were leading back to that reference state, with just a few metastable states found. Furthermore, one can observe that the trajectories converge to a well-defined relaxation path.

**Figure 2:**
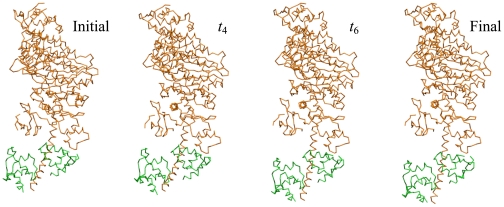
Relaxation motion in myosin V. Snapshots of the conformations of myosin V along the special relaxation path are shown. The essential light chain, displayed in green, is included into the model.

The red trajectory in Figures 1B,1C is for the relaxation starting from the nucleotide-free conformational state of myosin V (so that the mechanochemical motion following ATP binding is simulated). After a transient, this special trajectory joins the well-defined relaxation path. This functional trajectory is robust against perturbations, as shown by Figure 1D. Several snapshots of the conformation along this trajectory are shown in Figure 2 (see also Video S1).

The attractive path corresponds to a deep energy valley in the energy landscape of myosin V. Once this valley is entered, the conformational relaxation motion becomes effectively one-dimensional and characterized by a single mechanical coordinate. The profile of the elastic energy along the bottom of such energy valley determines the dependence of the elastic energy on the collective mechanical coordinate (see Methods).

**Figure 3:**
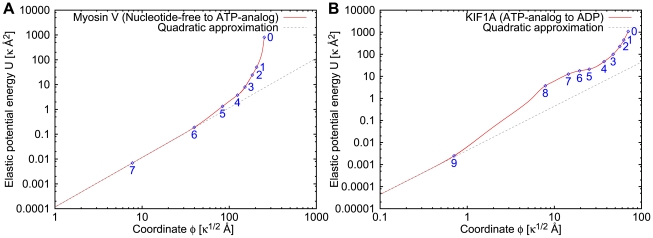
Profiles of elastic potential energy. (A) Myosin V and (B) KIF1A. Elastic energy 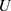 during transitions, corresponding to the trajectories shown by red lines in Figures 1 and 4, is plotted against coordinate 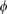. The dashed line shows 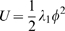, the quadratic approximation corresponding to the slowest normal mode (see Methods). Numbers correspond to time moments indicated in Figures 1C and 4C.

Figure 3A shows the dependence of the elastic energy along the special attractive relaxation path starting from the nucleotide-free state and leading to the ATP-bound state. Markers indicate positions along the trajectory in Figure 1C. For 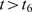, the elastic energy 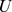 is approximately quadratic in terms of the mechanical coordinate 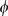, i.e. 
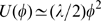
. Because 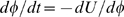, this implies that then 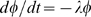 and the relaxation is exponential. Only within such harmonical neighborhood of the reference state, the normal mode description becomes applicable (see Methods for further discussion).

The dotted blue line in Figure 1D shows the direction of the distance changes corresponding to the slowest normal mode (see Methods). The nucleotide-free state of myosin V lies away from this direction and also outside of the harmonical neighborhood. The initial stage of the functional mechanochemical motion (until time 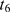) cannot be quantitatively analyzed in terms of the normal modes.

### Kinesin KIF1A

**Figure 4:**
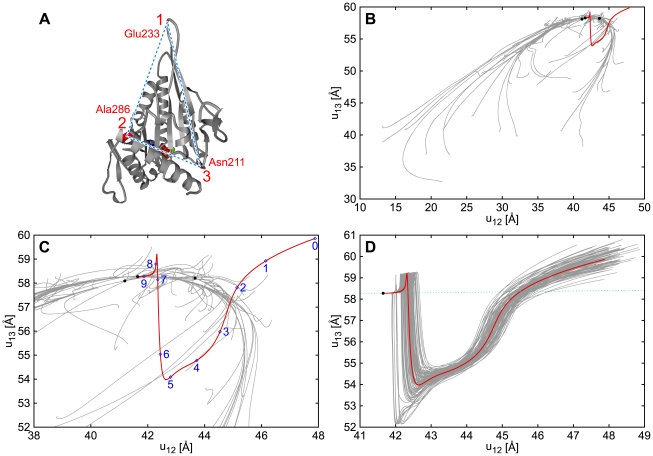
Relaxation paths of KIF1A. The ADP-bound structure has been used to construct the elastic network. The visualization labels are indicated in panel (A), and the relaxation paths are displayed in panels (B) to (D) in the same way as in Figure 1, panels (B) to (D), respectively. The initial condition for the red line is the ATP-bound state, so that this trajectory corresponds to the transition from the ATP-bound state to the ADP-bound state. Times 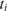 at points 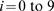 indicated in panel (C) are 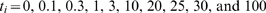, respectively.

The reference conformation for KIF1A is the ADP-bound state (PDB ID: 1I5S, with MgADP [32]). Relaxation starting from the initial condition, corresponding to the ATP(analog)-state (PDB ID: 1I6I, with MgAMPPCP as an ATP analog [32]) and from randomly generated initial conditions was considered. The elastic network has 320 particles and 2871 links. Only the residues whose 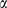-carbons are in both PDB data sets have been used. Visualization labels are Glu233, Ala286, and Asn211 (Figure 4A).

100 relaxation trajectories, starting from random initial conditions, are shown in Figures 4B,4C. The presence of an attractive relaxation path, corresponding to a deep energy valley, can be noticed.

The red lines in Figures 4B,4C display the special relaxation trajectory starting from the ATP-bound state. Surprisingly, we find that, in contrast to myosin V, this trajectory is *different* from the typical relaxation path. By applying small random initial perturbations to the initial ATP-bound state and integrating the dynamical equations, it can be demonstrated that this trajectory is, however, also stable with respect to the perturbations (Figure 4D). The dotted blue line in Figure 4D shows the direction of the distance changes in the slowest normal mode of KIF1A.

Thus, in KIF1A the deep energy valley leading to the reference ADP-bound state gets branched at some distance from it. The path corresponding to the functional mechanochemical motion from the ATP-bound state belongs to the side branch. Only at the final relaxation stage, in the immediate vicinity of the equilibrium, the valleys merge and the functional motion begins to coincide with the typical relaxation motion in this protein.

The branching of the energy valley is already an indication of strong nonlinearity in the relaxation dynamics. We have also determined the profile of the elastic energy 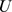 as a function of the mechanical coordinate 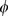 along the path connecting the ATP- and ADP-bound states (Figure 3B). The profile becomes quadratic only starting from time 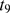, very close to the equilibrium reference state.

**Figure 5:**
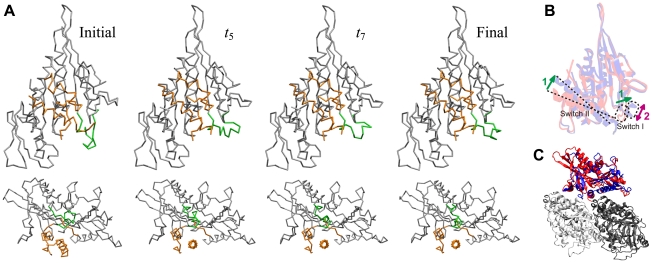
Relaxation motion in KIF1A. (A) Conformation snapshots (seen from two different viewpoints). Switch I and switch II regions are indicated in green and orange, respectively. (B,C) Schematic representation of the relaxation motion, observed in the simulation from the ATP-bound state (red) to the ADP-bound state (blue). In switch I region, as shown in panel (B), reconfiguration of the structure (2), i.e., transformation from a loop to an 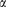-helix, occurs only after the sliding motion (1) is completed. For reference, relative positions of KIF1A and tubulin monomers (PDB ID: 2HXF and 2HXH [39]) are shown in panel (C).

Figure 5 shows snapshots of KIF1A along the special attractive relaxation path (see also Video S2). At the early relaxation stage (until 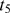), the relaxation motion represents a combination of the rotation of the switch II helix and of the sliding of the switch I loop. Relaxation at the end of such initial stage is apparently hindered, as revealed in the presence of a plateau in the dependence of the elastic energy on the mechanical coordinate in Figure 3B near 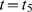. Only once the sliding is completed, further local structural reorganization, representing a transition from the loop to the 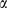-helix, becomes possible and is indeed observed approximately after time 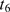.

## Discussion

The normal mode description is broadly used in structural studies of proteins. The analysis of thermal fluctuations and the interpretation of the respective experimental structural data are traditionally performed assuming that fluctuations are linear and, hence, correspond to thermal excitation of various normal modes (see, e.g., [3], [4]). The linear response of a protein macromolecule to structural perturbations, such as ligand binding, is an often used assumption [2]. To a large extent, the elastic-network analysis of ligand-induced macromolecular motions is based on determining normal modes in the elastic networks of the considered proteins (see, e.g., [7]). The patterns of atomic displacements in such normal modes are further compared with the experimentally measured atomic displacements in the same proteins that are induced by a change of the chemical state, such as binding of an ATP molecule [7], [14]–[17]. Large overlaps with only a few slowest normal modes are seen as the evidence for the applicability of the elastic-network ansatz, whereas the wide distribution of overlaps is considered as the indication that the elastic network description fails for a particular macromolecule. Specifically, strong overlaps between ligand-induced conformational changes and atomic displacements in the few slowest modes have been found for scallop myosin and 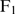-ATPase, while such overlaps were absent for kinesin KIF1A [18].

Our numerical investigations of elastic conformational motions in two motor proteins (myosin V and KIF1A) have revealed however that in both of them the nonlinearities play an essential role. While slow conformational relaxation motions in myosin can still be qualitatively characterized in terms of the normal modes, the normal mode description breaks down *completely* for KIF1A. The observed breakdown of the normal mode description does not however mean that conformational motions become irregular. We have seen that ordered and robust mechanochemical motions are characteristic for both protein motors, even though they cannot be described in terms of the linear response.

We want to emphasize that, when the dynamics is nonlinear, neither a single normal mode, nor a combination of many such modes can reproduce the motions. Thus, the normal mode description fails completely in this case and the problem is not that many normal modes must be taken into account. Actually, as we have shown, even for KIF1A, one normal mode would be sufficient to describe long-time relaxation within the harmonic domain — however, this domain is restricted to a tiny neighbourhood of the equilibrium state.

Thermal fluctuations have not been explicitly included into our dynamical ENM simulations. However, such fluctuations are effectively generating random conformational perturbations. In our study, relaxation processes starting from random conformational perturbations have indeed been considered.

In myosin V, one well-defined nonlinear conformational relaxation trajectory, leading to the equilibrium state, has been identified. Starting from an arbitrary initial conformation (but still without unfolding), rapid convergence to this special trajectory takes place. While the motion corresponding to the special attractive trajectory is initially nonlinear, it becomes harmonical later and a substantial part of the ordered conformational relaxation process is within the harmonic domain of the equilibrium state. Similar behavior has been previously noted by us [29] for scallop myosin and 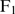-ATPase, but its detailed analysis has not yet been performed.

The situation is more complex for the monomeric kinesin KIF1A. Instead of a unique deep energy valley leading to the reference ADP-bound state, two such valleys, both leading to the equilibrium state, are present. These valleys correspond to two kinds of ordered conformational motions possible in the protein.

The first of them is relatively wide and, when thermal conformational fluctuations are excited, they would typically proceed along it. However, the conformational relaxation motion starting from the ATP-bound state follows a different path, which corresponds to the second energy valley branching from the typical fluctuation path already at very small deviations from the equilibrium state. Note that the branching takes place as the change in the distance between the molecular labels Glu233 and Ala286 is still less than an angstrom, which is much smaller than the intensity of typical thermal fluctuations for such a distance. Thus, the nonlinear effects in KIF1A are strong even for the typical thermal fluctuations.

Remarkably, such second relaxation path is also stable with respect to perturbations, i.e. structurally robust. Our numerical investigations reveal that motion along this path can be divided into two *qualitatively different* stages. At the first of them, sliding of the switch I loop is observed, whereas at the second stage a transition from the loop to the 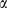-helix is realized. Structural reorganization, corresponding to this transition, is not possible until the sliding motion is completed, lifting a restriction through the backbone chain. Recent crystallographic studies suggest that the switch I loop/helix plays an important role in control of the motor function through interaction with 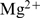 and switch II [33].

Thus, in contrast to myosin, a single ATP binding event induces in KIF1A a complex, but ordered conformational motion characterized by two qualitatively different consequent phases. As we conjecture, this special dynamical property of KIF1A may be needed for the processive motion of this single-headed molecular motor [24].

In myosin V, conformational motions driven by random thermal fluctuations are similar in their properties to the relaxation motion from the nucleotide-free state. This may facilitate exploitation of such fluctuation motions for the motor operation, as suggested by recent single-molecule experiments [34]. In KIF1A, where the energy valley splits into two branches, typical thermal conformational fluctuations are qualitatively different from the relaxation motion starting from the previous ATP-bound state. The latter motion is entropically hindered for thermal fluctuations and cannot be reversed through them. This may turn out to be important for the understanding of the operation of the monomeric kinesin as a molecular motor. Latest experimental techniques permit simultaneous observation of stepping motion and conformational changes of a motor [35]. The coarse-grained modeling, including our present study, can contribute further suggestions for the design (e.g., by determining positions for fluorescent labeling) of such experiments.

Finally, we note that our study has been based on the elastic network approximation for proteins. More detailed descriptions, such as, e.g., G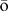-like models, can also be used to consider conformational relaxation processes [36]. We expect that similar behavior will then be observed.

## Methods

### Elastic network models

In this study, we employ elastic network models where material points are connected by a set of elastic springs [8]–[11]. Each particle corresponds to a residue in the considered protein. The equilibrium positions 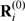 of the particles are determined by the locations of 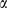-carbon atoms in the reference state of the protein, taken from the PDB database. Two particles in a network are connected by an elastic spring if at equilibrium the distance 
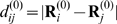
 between them is less than a certain cutoff length 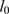. The natural length of an elastic link is equal to the equilibrium distance 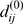. The cutoff distance 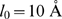 has been used in our study.

The elastic forces obey the Hooke law and all springs have the same stiffness constant 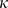. Elastic torsion effects are not included. Thus, the force acting on particle 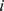 is
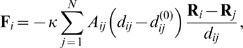
(1)where 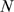 is the total number of particles in the network, 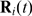 is the actual position of the particle 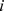 and 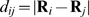 is the actual distance between two particles 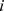 and 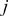. The adjacency matrix of the network is defined as having 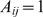 if 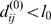 and 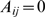 otherwise. The total elastic energy of the network is
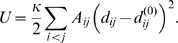
(2)


Because slow conformational dynamics of proteins in the solvent is considered, the motions are overdamped (see [25]) and the velocity of a particle is proportional to the force acting on it, i.e. 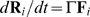 where 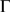 is the mobility. We assume that the mobilities of all particles are the same. Hydrodynamical effects are neglected (they can be however incorporated into the elastic network models as shown in ref. [37]).

Explicitly, the relaxation dynamics is described by a set of differential equations:
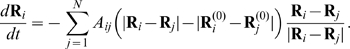
(3)Here, time is rescaled and measured in units of 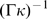. Hence, the relaxation dynamics of a network is completely determined by its pattern of connections (matrix 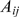) and the equilibrium distances 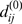 between the particles. Equations (3) were integrated to determine conformational relaxation motions.

### Simulations

To prepare random initial conditions, the following procedure has been employed. Random static forces 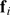, acting on all particles in the network have been independently generated with the constraint that 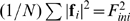. The equations of motion were integrated in the presence of such static forces for a fixed time 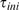. The conformation which was thus reached has been then used as the initial condition for the relaxation simulation. The parameters were 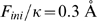, 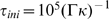 for myosin V and 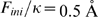, 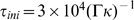 for KIF1A. With these parameter values, relatively large overall deformations (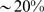 typical) could be reached, while still avoiding unfolding. In the deformed states, the lengths of the links did not exceed 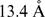 for myosin V and 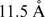 for KIF1A.

When relaxation from specific conformations has been considered, initial positions of all particles were allocated according to the respective PDB structures. When robustness of a relaxation path starting from a specific conformation was investigated, the initial condition was prepared by randomly shifting the positions of all particles with respect to their locations in that conformation with a certain root-mean-square displacement 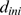. We have chosen 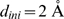 for myosin V and 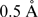 for KIF1A.

To visualize conformational motions, three particles labeled as 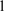, 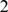 and 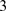 were chosen and the distances 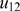 and 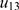 were monitored in the simulations. Thus, the relaxation motion was represented by a trajectory on the plane 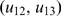.

The choice of the visualization labels is essentially arbitrary. In a simulation, motions of all residues were traced (see, e.g., Videos S1 and S2) and different residues could be selected for a specific visualization. If a molecule has a low-dimensional attractive relaxation manifold, this is a property of the respective dynamical system and it cannot depend on the visualization method. When selecting the labels, one should only pay attention to the fact that the distances between them should significantly vary during the relaxation process. If, by chance, two labels belonging to the same stiff domain in a protein have been taken, the distance between them would remain almost constant, so that such a choice would be inconvenient. When the normal mode description approximately holds and, furthermore, relaxation is well described by a few slowest modes, one can choose the labels so that the distances between them reveal variations characteristic for the first few normal modes. Such selection was previously made [29] for scallop myosin and 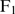-ATPase, and it has been adopted in the present study for myosin V. For KIF1A, the labels have been chosen in such a way that motions in switch I and switch II regions are well resolved.

### Profiles of elastic potential energy

The collective mechanical coordinate 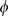 along a relaxation path was defined by requiring that its dynamics obeys the equation 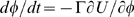 and that 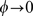 as 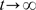. Multiplying both parts of this equation by 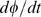, we find that it is equivalent to the equation
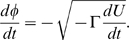
(4)Equation (4) can be used to determine the coordinate 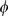 along a given relaxation trajectory and the dependence of the elastic energy 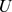 on this coordinate.

For each point along the trajectory, the time 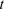 when it is reached in the relaxation process is known. Moreover, the actual network configuration corresponding to this point is also known from the simulation. Therefore, for each point specified by time 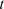 the respective elastic energy 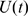 is determined. The mechanical coordinate 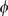, reached at time 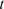, is given by the integral
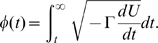
(5)


### Normal mode description

We provide a summary of the results on the normal mode description of conformational relaxation processes. If deviations 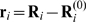 from the reference conformation are small for all particles, the nonlinear equations (3), describing conformational relaxation of an elastic network, can be linearized:
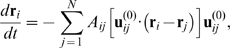
(6)where 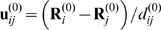.

Equations (6) can be written in the matrix form as
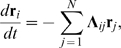
(7)where 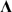 is the 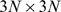 linearization matrix:
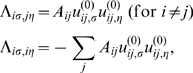
(8)where 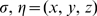.

The general solution of these linear differential equations is given by a superposition of 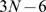 exponentially decaying normal modes, i.e.
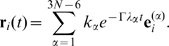
(9)Here, 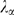 and 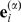 are the eigenvalues and the eigenvectors of the linearization matrix, i.e.
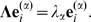
(10)This matrix has 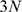 eigenvalues, but 6 of them must be zero, corresponding to free translations and rotations of the entire network.

Generally, all normal modes are initially present. As time goes on, first the normal modes with the larger eigenvalues 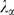 decay. In the long time limit, relaxation is characterized by the soft modes corresponding to the lowest eigenvalues.

**Figure 6:**
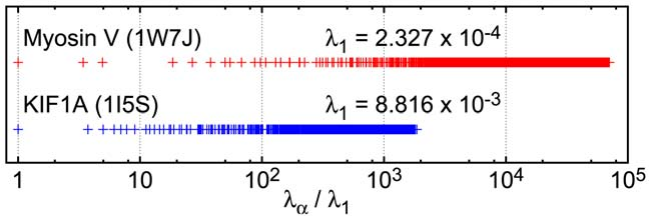
Eigenvalue spectra of the elastic network models. Eigenvalues 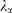 in the normal mode description (eqn. (10)), normalized to the lowest non-zero eigenvalue 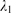, are shown. There is a significant gap between the lowest and the second lowest modes.

Figure 6 shows the computed eigenvalue spectra of myosin V and KIF1A. The eigenvalues are normalized to the lowest nonzero eigenvalue 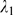 and the logarithmic representation is chosen.

Note that in both motor proteins a significant gap, separating the soft mode from the rest of the spectrum, is present. This means that, in the linear approximation, long-time relaxation in these proteins is effectively characterized by a single degree of freedom, representing the amplitude of the soft mode. The pattern of displacements of particles (i.e., residues) from the reference positions is determined by the eigenvector 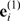 of the soft mode.

In the plane 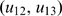 of the distances between the labels 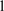, 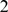 and 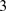, used by us for the visualization of conformational motions, the exponential relaxation motion corresponding to the soft mode should proceed along the direction defined by the vector with the components 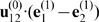 and 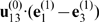. Such directions are indicated by dotted blue lines in Figure 1D and Figure 4D.

When relaxation is reduced to a single soft mode, the elastic potential is quadratic in terms of the mechanical coordinate, i.e. 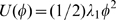.

Note that the representation of the relaxation process as a superposition (9) of normal relaxation modes holds only in the harmonic domain, i.e. when linearization (6) of full nonlinear relaxation dynamics equations (3) is valid. If dynamics is nonlinear and the linearization does not hold, relaxation dynamics cannot be viewed *at all* as a superposition of any normal modes. Whether just one normal mode or many of them should be included into a description of long-time relaxation dynamics is determined by the properties of the eigenvalue spectrum and not related to the possible invalidity of the harmonic approximation.

As an extension, iterative normal mode analysis has been proposed [21], [38]. This method is applied to obtain an optimal sequence of conformational states, transforming an initial given conformation into a target conformation, which may be known with a low resolution or only partially, and thus to reconstruct missing details of that structure. Each next conformation in the sequence is obtained by making a step into the direction maximizing similarity with the target, restricted however to a superposition of a certain number of the lower normal modes. At the next iteration step, the previous conformation is chosen as a new reference state and a new set of normal modes is determined. This prediction method is useful and provides valuable results, e.g., in the refinement of low-resolution structures from electron microscopy [38]. It should be however emphasized that the sequence of conformational states yielded by such a method is generally different from the path along which conformational relaxation from the target to the reference state would proceed. Even in the normal mode approximation, dynamics of conformational relaxation depends not only on the eigenvectors, but also — and very significantly — on the eigenvalues of normal modes. Generally, the next iteration state in this method would not be the next conformation along the actual relaxation path. This difference can be clearly demonstrated by considering the example of KIF1A. The conformational relaxation path transforming the initial ATP-bound state into the (equilibrium) ADP-bound state is non-monotonous (Figure 4). It proceeds via intermediate states (particularly of the switch I region) which cannot be obtained by gradual interpolation maximizing similarity of the structures along the optimization path.

## Supporting Information

Video S1 The motion of myosin V along the special relaxation path. t = 0 to 2000.
(1.25 MB MOV)

Video S2 The motion of KIF1A along the special relaxation path. t = 0 to 50.
(0.76 MB MOV)
